# Synthesis, Crystal Structure, DFT, and Anticancer Activity of Some Imine-Type Compounds via Routine Schiff Base Reaction: An Example of Unexpected Cyclization to Oxazine Derivative

**DOI:** 10.3390/molecules28124766

**Published:** 2023-06-14

**Authors:** Jamal Lasri, Naser E. Eltayeb, Saied M. Soliman, Ehab M. M. Ali, Sultan Alhayyani, Abdullah Akhdhar

**Affiliations:** 1 Department of Chemistry, Rabigh College of Science and Arts, King Abdulaziz University, P.O. Box 344, Jeddah 21589, Saudi Arabia; 2 Department of Chemistry, Faculty of Science, Alexandria University, Ibrahimia, Alexandria 21321, Egypt; 3 Department of Biochemistry, Faculty of Science, King Abdulaziz University, Jeddah 21589, Saudi Arabia; 4 Department of Chemistry, College of Science, University of Jeddah, Jeddah 21959, Saudi Arabia

**Keywords:** imine, oxazine, DFT, Hirshfeld, intermolecular interactions, anticancer

## Abstract

The synthesis, characterization, and anticancer properties of three imine-type compounds **1**–**3** and an unexpected oxazine derivative **4** are presented. The reaction of *p*-dimethylaminobenzaldehyde or *m*-nitrobenzaldehyde with hydroxylamine hydrochloride afforded the corresponding oximes **1**–**2** in good yields. Additionally, the treatment of benzil with 4-aminoantipyrine or *o*-aminophenol was investigated. Routinely, the Schiff base (4*E*)-4-(2-oxo-1,2-diphenylethylideneamino)-1,2-dihydro-1,5-dimethyl-2-phenylpyrazol-3-one **3** was obtained in the case of 4-aminoantipyrine. Unexpectedly, the reaction of benzil with *o*-aminophenol proceeded with cyclization to produce the 2,3-diphenyl-2*H*-benzo[*b*][1,4]oxazin-2-ol **4**. The structures of compounds **3** and **4** were unambiguously determined by single crystal X-ray diffraction. Hirshfeld analysis of molecular packing revealed the importance of the O…H (11.1%), N…H (3.4%), C…H (29.4%), and C…C (1.6) interactions in the crystal stability of **3**. In the case of **4**, the O…H (8.8%), N…H (5.7%), and C…H (30.3%) interactions are the most important. DFT calculations predicted that both compounds have a polar nature, and **3** (3.4489 Debye) has higher polarity than **4** (2.1554 Debye). Different reactivity descriptors were calculated for both systems based on the HOMO and LUMO energies. The NMR chemical shifts were calculated and were found well correlated with the experimental data. HepG2 growth was suppressed by the four compounds more than MCF-7. The IC_50_ values of **1** against HepG2 and MCF-7 cell lines were the lowest, and it is considered the most promising candidate as an anticancer agent.

## 1. Introduction

The transformation of carbonyl functional groups into imine derivatives is a well-known conversion in organic synthesis. Oximes are imine compounds having the general formula HO–N=CR^1^R^2^ and are described as a significant class of compounds with diverse applications, not only for the protection of carbonyl functionality but also for the purification and/or characterization of compounds bearing a carbonyl group [1,2]. Oxime derivatives have several applications in medicine and can be employed as antidotes for nerve agents [3]. They might function as cholinesterase inhibitors as well. Drugs that suppress cholinesterase are used to treat dementia and Alzheimer’s symptoms [4,5]. Oximes are employed as intermediates in the preparation of caprolactam, which is a precursor of nylon 6 [6]. The transformations to nitriles [7], nitro products [8,9], nitrones [10], amines [11], and aza heterocycles [12] are some of the synthetic uses of oximes. It has been reported that oximes are also useful reagents for selective activation [13] and widely employed as intermediates for the synthesis of amides by Beckmann rearrangement [14,15], fungicides, and herbicides [16]. Oximes are valuable reactants containing the O–N=C fragment [17], which can be added to the nitrile functional group to produce several *nitrogen*-*containing compounds*, e.g., iminoacylate [18,19,20], amidines [21], carboxamides [22], phthalocyanines [23], or 1,3,5-triazapentadiene species [24]. Lasri et al. reported the synthesis and characterization of ferrocenecarboxaldehyde oxime and its use for the anionic methyl blue dye removal from wastewater [25]. On the other hand, Lasri et al. also reported the synthesis, crystal structure, DFT, and biological study of various aldoximes, i.e., *E*-1-naphthaldehyde oxime, *E*-9-phenanthrenecarboxaldehyde oxime, *E*-pyrene-1-carbaldehyde oxime, and *E*-2-naphthaldehyde oxime [26,27].

The substance 4-aminoantipyrine belongs to the family of phenazones, useful heterocyclic compounds known for their important role in organic chemistry [28] and various biological applications [29,30]. Benzil (i.e., Bz_2_, known as 1,2-diphenylethane-1,2-dione) is a common building block in organic synthesis that is also reported to be a potent inhibitor of mammalian carboxylesterase enzymes [31]. On the other hand, the condensation reaction of 2-aminoethanol, *o*-aminophenol, and their related compounds, containing N or S atoms instead of O, with *α*-diketones, has been the topic of intensive research work [32,33,34].

In the light of the above findings, we report herein the synthesis and characterization of the two aryl aldoximes *p*-dimethylaminobenzaldoxime **1** and *m*-nitrobenzaldoxime **2**, as well as the Schiff base **3** of benzil and 4-aminoantipyrine and the unexpected hemiacetal oxazine cycloadduct **4** of benzil and *o*-aminophenol. The X-ray crystal structures and DFT studies for **3** and **4** are also described. In addition, the anticancer potentials of the studied compounds **1**–**4** were investigated.

## 2. Results and Discussion

### 2.1. Synthesis and Characterization of Aryl Aldoximes **1** and **2**

**Scheme 1:**
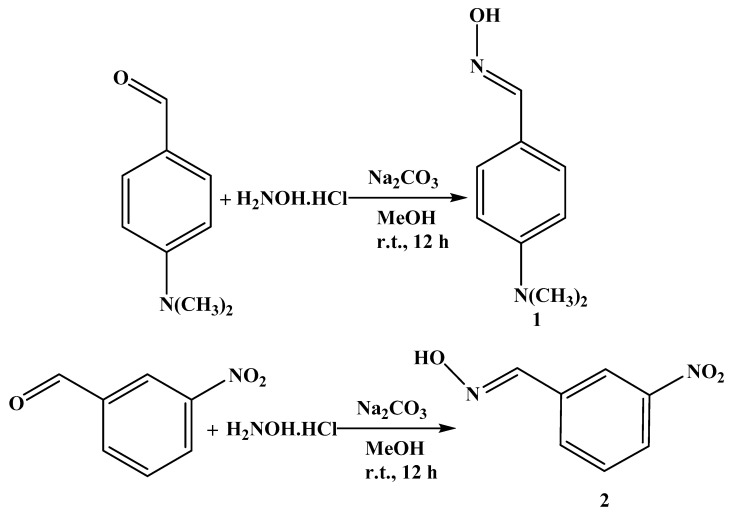
Synthesis of aryl aldoximes **1** and **2**.

The aryl aldoximes *p*-dimethylaminobenzaldoxime **1** and *m*-nitrobenzaldoxime **2** were synthesized, in ca. 90% yield, by treatment of *p*-dimethylaminobenzaldehyde or *m*-nitrobenzaldehyde, respectively, with sodium carbonate and hydroxylamine hydrochloride in MeOH at room temperature (Scheme 1).

Compounds **1** and **2** were characterized by FT-IR, ^1^H, and ^13^C{^1^H} NMR spectroscopy and elemental analyses. The FT-IR spectra of *p*-dimethylaminobenzaldoxime **1** and *m*-nitrobenzaldoxime **2** showed the characteristic bands at wavenumbers 3246 and 3298 cm^−1^ (O−H) and 1606 and 1617 cm^−1^ (C=N), respectively, which confirm the formation of the aryl aldoximes **1** and **2**. In the ^1^H NMR spectra of **1** and **2**, we detected the absence of the signal of the aldehyde functional group at ca. 10 ppm, and new signals at 7.98 and 8.36 ppm due to the imine protons C*H*=N of **1** and **2**, respectively, were observed. Moreover, in the ^13^C{^1^H} NMR spectra of **1** and **2**, the signal of the aldehyde at ca. 190 ppm was not detected, and new signals at 150.3 and 147.2 ppm due to the oxime carbons *C*=NOH of **1** and **2**, respectively, were observed, confirming the formation of the aryl aldoximes **1** and **2** (see Section 3).

### 2.2. Synthesis and Characterization of Compounds **3** and **4**

**Scheme 2:**
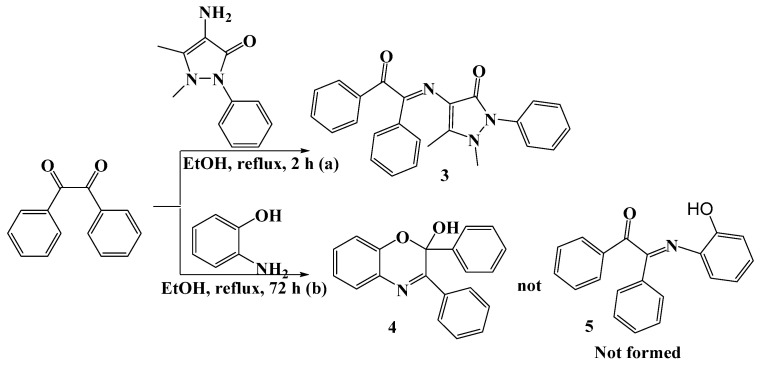
Synthesis of compounds **3** and **4**.

The corresponding (4*E*)-4-(2-oxo-1,2-diphenylethylideneamino)-1,2-dihydro-1,5-dimethyl-2-phenylpyrazol-3-one **3** and 2,3-diphenyl-2*H*-benzo[*b*][1,4]oxazin-2-ol **4** were synthesized, in ca. 88% yield, by treatment of benzil with 4-aminoantipyrine or *o*-aminophenol in refluxing EtOH (Scheme 2).

Compounds **3** and **4** were characterized by FT-IR, ^1^H, and ^13^C{^1^H} NMR spectroscopy, elemental analyses, and single crystal X-ray diffraction. The FT-IR spectrum of compound **3** showed the characteristic band at 1663 cm^−1^ (C=N), which confirms the presence of the imine moiety. In the ^1^H NMR spectrum of **3**, the new signals at 2.32 and 2.94 ppm due to the methyl protons were observed, and the signals in the range of 7.1 to 7.8 ppm due to the phenyl protons were also detected. Moreover, in the ^13^C{^1^H} NMR spectrum of **3**, the new signals at 158.7 and 162.1 ppm confirm the presence of the amido (*C*(N)=O) and imino (C=N) moieties, respectively. The presence of the carbonyl (C=O) signal at 194.5 ppm indicates that the condensation occurs to only one carbonyl group of benzil (see Section 3).

The FT-IR spectrum of compound **4** showed the characteristic bands at 3240 (OH), 1667 (C=N), 1589 (C=C), and 1092 (C-O), which confirms the formation of cycloadduct **4**. In the ^1^H NMR spectrum of **4**, a broad signal at 3.82 ppm due to the O*H* proton was observed, and the signals in the range of 6.5 to 7.9 ppm due to the phenyl protons were also detected. Moreover, in the ^13^C{^1^H} NMR spectrum of **4**, the new signal at 159.9 ppm confirms the presence of the imino (C=N) moiety. The presence of the signal at 94.7 ppm due to the *hemiacetal* carbon (O*C*O) confirms the nucleophilic attack of the hydroxyl group of *o*-aminophenol on the carbonyl group of benzil, followed by a ring closure to produce the cycloadduct 2,3-diphenyl-2*H*-benzo[*b*][1,4]oxazin-2-ol **4** (see Section 3).

### 2.3. X-ray Structure Description

**Figure 1:**
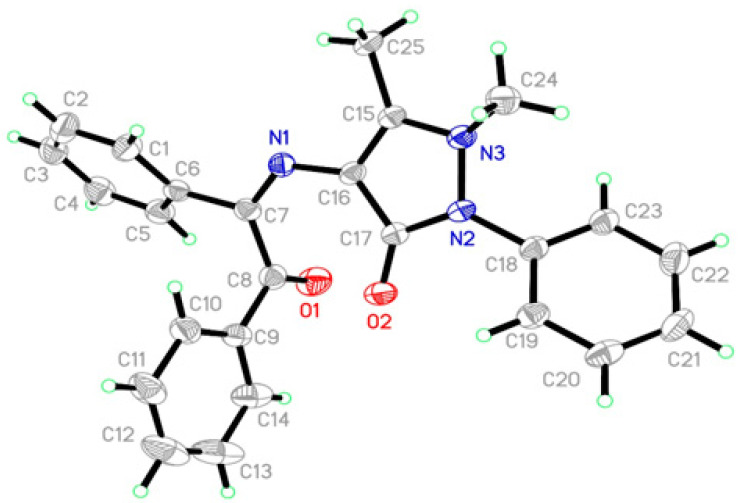
Crystal structure of molecule **3**.

In the structure of compound **3** (Figure 1), the dihedral angles between the three phenyl rings identified by ***A*** (C1–C6), ***B*** (C9–C14), ***C*** (C18–C23), and pyrazole ring ***D*** (C15/C16/C17/N2/N3) are ***A/D*** = 49.4(2) °, ***B/D*** = 81.5(2) °, and ***C/D*** = 45.8(2)°, respectively. This result showed that the phenyl rings ***A***, ***B*,** and ***C*** are out of the plane of pyrazole ring ***D***. Analysis of bond lengths revealed that C16–N1 is 1.403(5) Å and C7=N1 is 1.283(5) Å, with the C–N and C=N bond lengths indicating single and double bonds, respectively, which is similar to Schiff base compounds [35,36]. The torsional angles of C16/N1/C7/C6 and C16/N1/C7/C8, which are connected to the phenyl rings ***A*** (C1–C6) and ***B*** (C9–C14) with pyrazole ring ***D*** (C15/C16/C17/N2/N3), are 178.4(3) ° and −6.7(6) °, respectively. In the molecular structure, the crystal packing is stabilized by intermolecular O–H…N and C–H...O hydrogen bonds and C–H…π short interactions.

**Figure 2:**
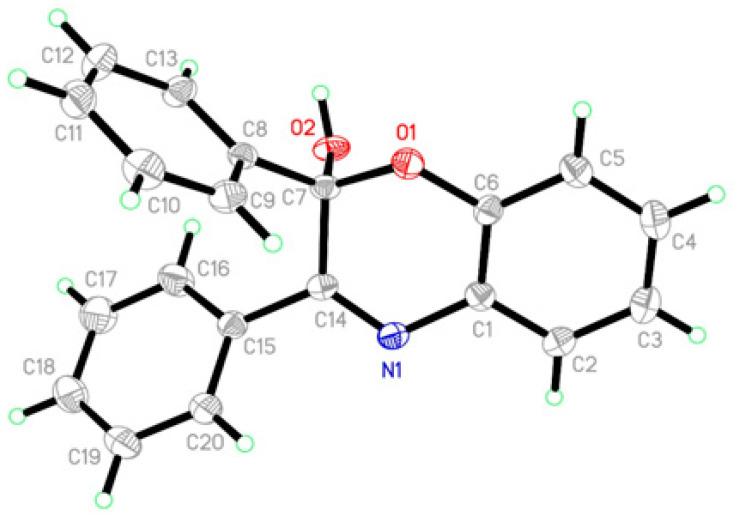
Crystal structure of molecule **4**.

In the structure of compound **4** (Figure 2), the dihedral angles between the three phenyl rings identified by ***A*** (C1–C6), ***B*** (C8–C13), and ***C*** (C15–C20) are A/B = 85.08(10)°, A/C = 53.86(12)°, and B/C = 64.86(12)°, respectively. This result shows that the phenyl rings ***B*** and ***C*** are out of the plane of phenyl ring ***A***. Analysis of bond lengths revealed that C1–N1 is 1.422(3) Å and C14=N1 is 1.280(2) Å, with the C–N and C=N bond lengths indicating single and double bonds, respectively, which is similar to Schiff base compounds [35,36]. In the molecular structure, the crystal packing is stabilized by intramolecular C–H...O, intermolecular O–H...N hydrogen bonds, and C–H…π short interactions.

### 2.4. Analysis of Molecular Packing

**Figure 3:**
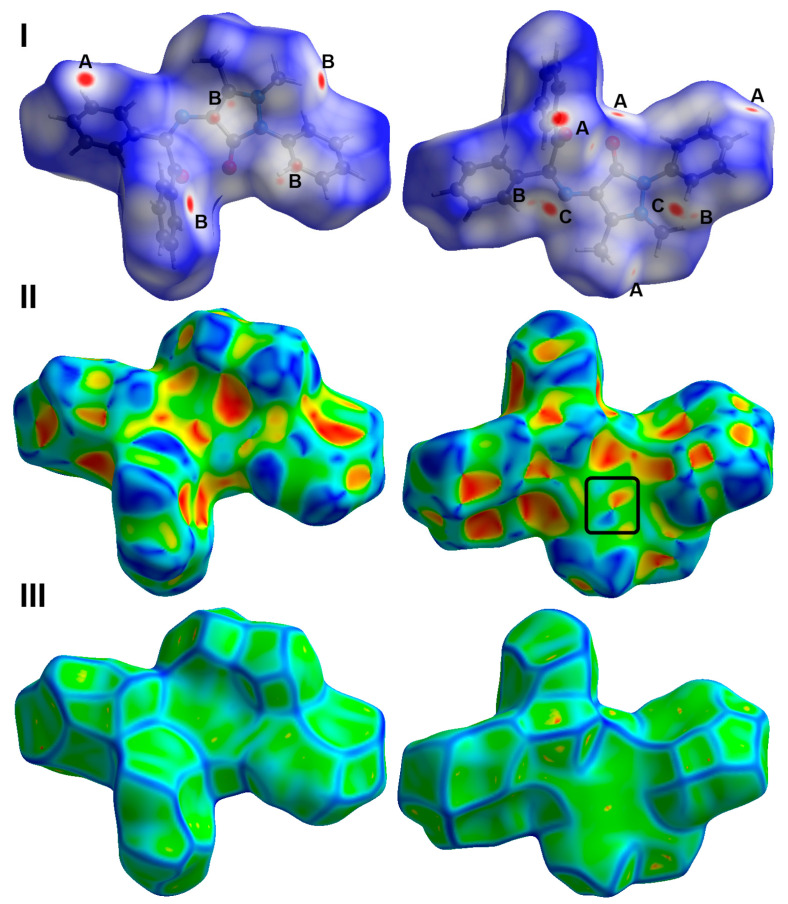
Hirshfeld surfaces: (**I**) d_norm_, (**II**) shape index, and (**III**) curvedness of **3**. A: O…H, B: N…H, C: C…H/C…C contacts.

**Figure 4:**
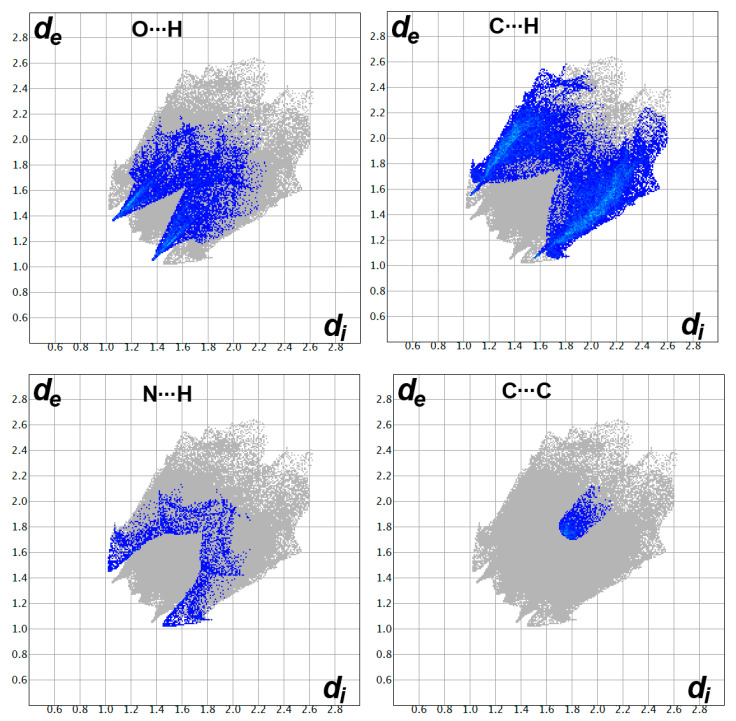
Fingerprint plots for the important interactions in **3**.

Hirshfeld surface analysis of **3** was performed in order to further analyze the molecular packing. The Hirshfeld surfaces are shown in Figure 3. All red spots in the d_norm_ map represent regions where there are short distance contacts, and those are considered significant for molecular packing. The most important contacts are O…H, N…H, C…H, and C…C interactions. Their percentages are 11.1, 3.4, 29.4, and 1.6%, respectively. The contact distances of these short contacts are depicted in Table 1. The majority of these short contacts appeared as sharp spikes in the fingerprint plots (Figure 4), which sheds light on the importance of these intermolecular contacts in the molecular packing of **3**.

**Figure 5:**
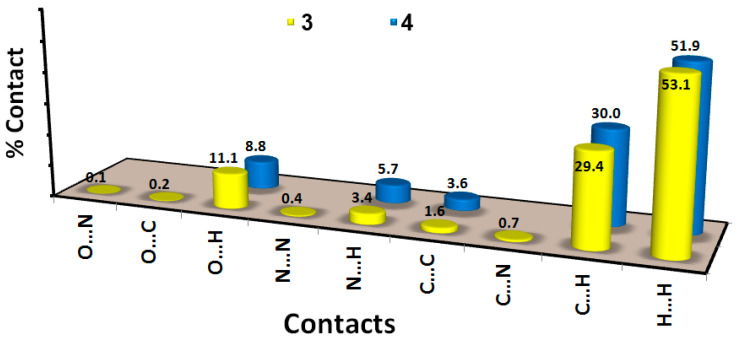
Intermolecular interactions in **3** and **4**.

As clearly seen from Figure 5, there are many different contacts controlling the molecular packing of this compound. The most abundant interaction in the crystal structure of **3** is the H…H contacts, which contribute to more than half the intermolecular interactions. The H…H contact percentage is 53.1%. Similarly, the contribution of this interaction in the molecular packing is 51.9% for compound **4**.

**Figure 6:**
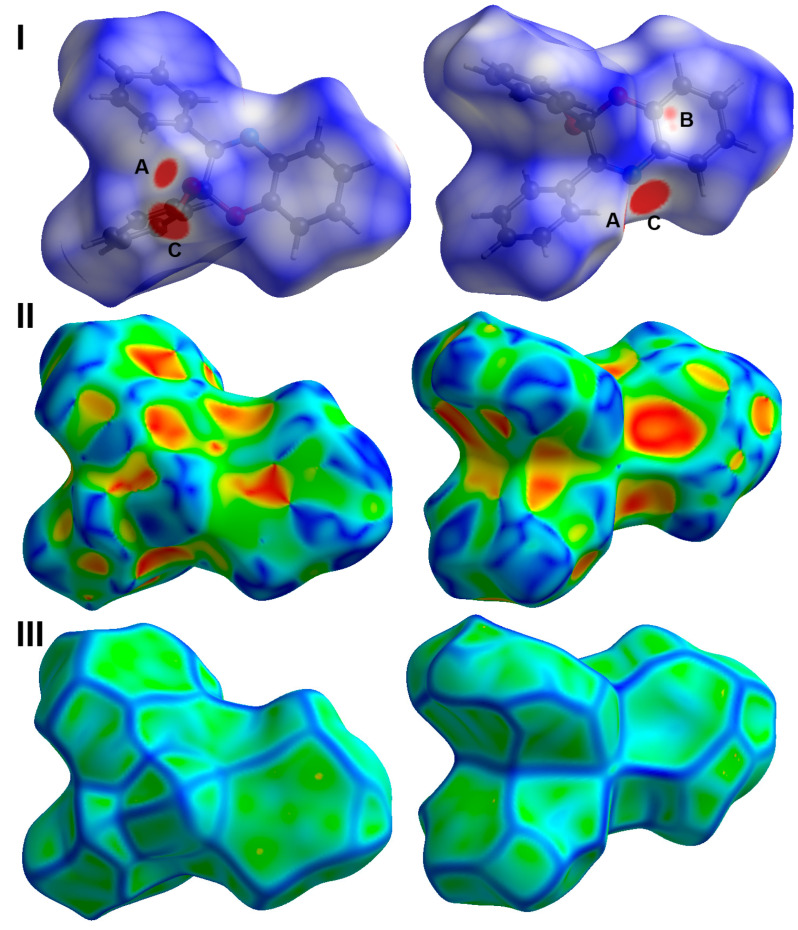
Hirshfeld surfaces: (**I**) d_norm_, (**II**) shape index, and (**III**) curvedness of **4**. A: O…H, B: N…H, C: C…H/C…C contacts.

**Figure 7:**
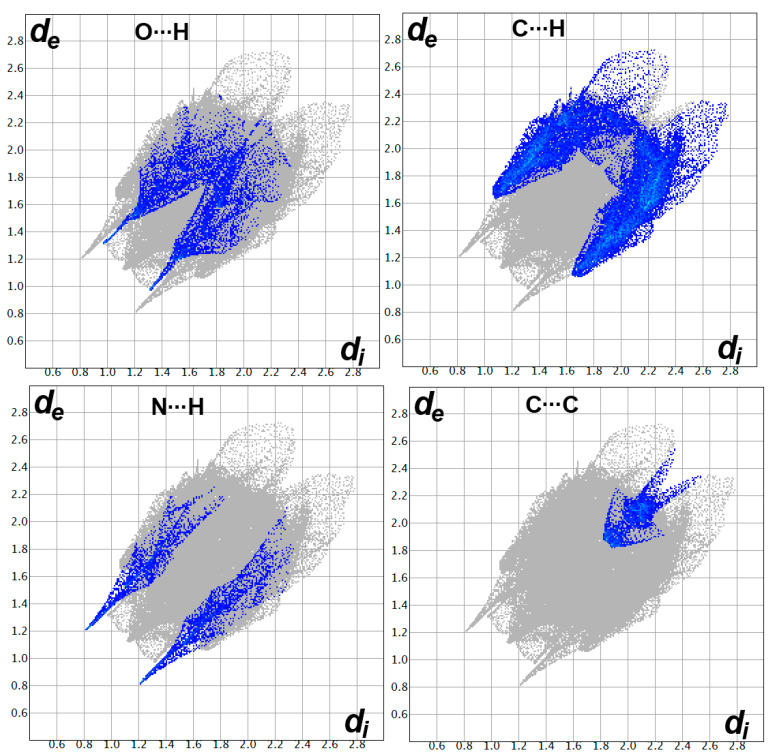
Fingerprint plots for the important interactions in **4**.

Similarly, the Hirshfeld surfaces of **4** shown in Figure 6 revealed the importance of the O…H, N…H and C…H interactions in the molecular packing of this compound. These interactions contributed by 8.8, 5.7, and 30.0%, respectively, from the whole contacts detected in **4** (Figure 7). Although there is a higher percentage of the C…C contacts in this compound (3.6%) compared to **3**, there are no significant C…C interactions detected in **4**, where all appeared as the blue region in the d_norm_. Additionally, the importance of the π-π stacking interactions in the former is further revealed from the shape index with red/blue triangles and curvedness, with a flat green area where these features are totally absent in the latter. The only contacts that appeared as red spots in d_norm_ and sharp spikes in the fingerprint plots are the O…H, N…H, and C…H interactions (Figure 6). The interaction distances are summarized in Table 1.

A clear difference between the two compounds is the shorter O…H and N…H contacts in **4** compared to **3**. In the former, the hydrogen-acceptor distance of the C-H…O interaction is 2.284 Å (O2…H20), while the corresponding values range from 2.420 Å (O1…H2) to 2.557 Å (O1…H25B) in the latter. In addition, the N1…H1 (2.028 Å) contact in **4** occurred at a shorter distance than the N1…H23 (2.482 Å) in **3**. In contrast, the C6…H3 contact (2.718 Å) in **4** is longer than those found in **3**. In the latter, the shortest C…H interaction is C7…H23 (2.608 Å).

### 2.5. DFT Studies

**Figure 8:**
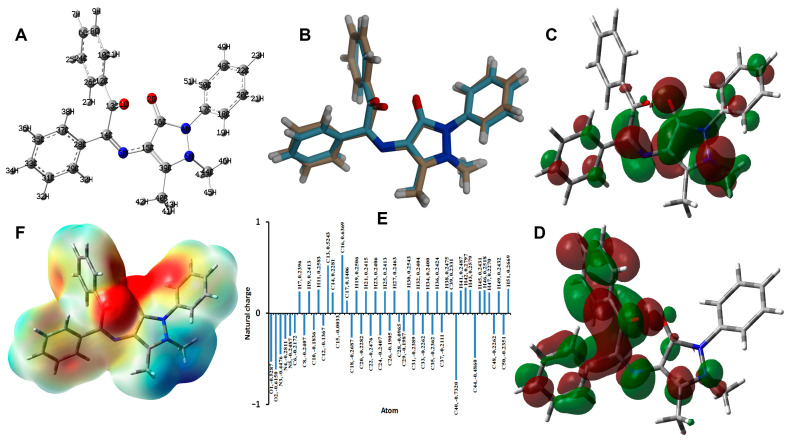
Optimized geometry (**A**), overlay of the optimized/experimental structures (**B**), HOMO (**C**), LUMO (**D**), natural charge (**E**), and MEP (**F**) for **3**.

**Figure 9:**
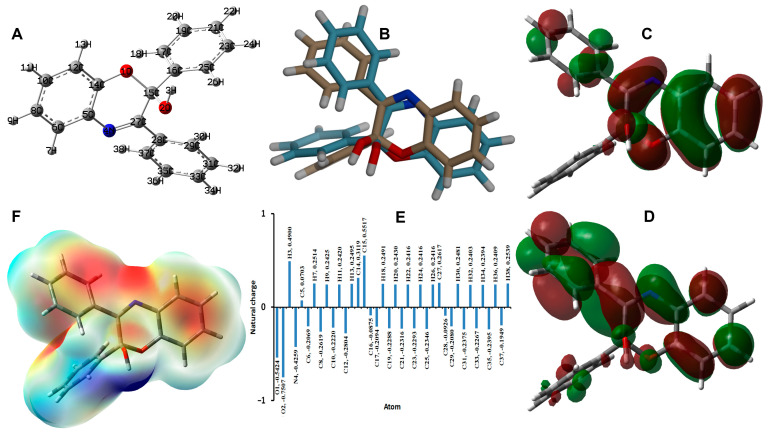
Optimized geometry (**A**), overlay of the optimized/experimental structures (**B**), HOMO (**C**), LUMO (**D**), natural charge (**E**), and MEP (**F**) for **4**.

The B3LYP method is one of the most common and accurate methods for predicting the molecular structure and electronic and spectroscopic properties of molecular systems [37,38,39]. The structures of **3** and **4** were optimized using the B3LYP/6-31G(d,p) method. The calculated and experimental structures along with their overlay are shown in Figure 8 and Figure 9, respectively, while the calculated geometric parameters are listed in Tables S1 and S2 (Supplementary Materials). There are some deviations between the calculated and experimental data that could be attributed to the well-known fact that the experimental structure belongs to a molecule in the crystal that is affected by the intermolecular interactions with the neighboring molecules, while the calculated structure is for a single isolated molecule in the gas phase.

For **3**, the molecular structure comprised different charged regions, as shown from the natural charge analysis and the map of electron density over electrostatic potential shown in Figure 8. The negatively charged regions are related to the oxygen atoms of the carbonyl groups. As a result, the molecule of **3** has a net dipole moment of 3.4489 Debye. Based on the natural charge analysis, the two oxygen atoms of the carbonyl groups have high negative charges, while the hydrogen atoms are the most positive atomic sites along with the carbonyl carbon. These regions are mapped with red and blue colors for the most negative and most positive regions, respectively. Another electronic feature that is related to molecular reactivity is the frontier molecular orbitals HOMO and LUMO. Their energies are calculated to be −5.4399 and −1.221 eV, respectively, leading to ionization potential (I = −E_HOMO_), electron affinity (A = −E_LUMO_), chemical potential (μ = −(I + A)/2), hardness (η = (I − A)/2), and electrophilicity index (ω = μ^2^/2η) [40,41,42,43,44,45] of 5.4399, 1.6221, −3.5310, 3.8178, and 1.6329 eV, respectively. The distribution of the HOMO and LUMO is delocalized over the conjugated π-system, indicating a π-π intramolecular charge transfer due to this lowest energy excitation, where the HOMO to LUMO excitation energy is 3.8178 eV.

Similarly, the optimized structure and the different electronic parameters of **4** are presented in Figure 9. In addition, the most negative atomic sites are the O-atoms of hydroxyl and cyclic ether groups, respectively. Their natural charges are calculated to be −0.7505 and −0.5424 e, respectively. The carbon atoms bonded to these O-sites are the most positive. Their natural charges are 0.5517 and 0.3119, respectively. Moreover, the hydroxyl proton possesses a high positive charge of 0.4900. The MEP map revealed the high electron density related to the oxygen atoms and the high positive charge density at the OH proton. The calculated dipole moment is 2.1554 Debye, indicating less polarity compared to **3**. In addition, the HOMO and LUMO energies are calculated to be −5.8442 and −1.5252 eV, respectively. The energy separation between the HOMO and LUMO levels is 4.3190 eV. Moreover, the ionization potential, electron affinity, chemical potential, hardness, and electrophilicity index are calculated to be 5.8442, 1.5252, −3.6847, 4.3190, and 1.5718 eV, respectively.

### 2.6. Calculated NMR Spectra

**Figure 10:**
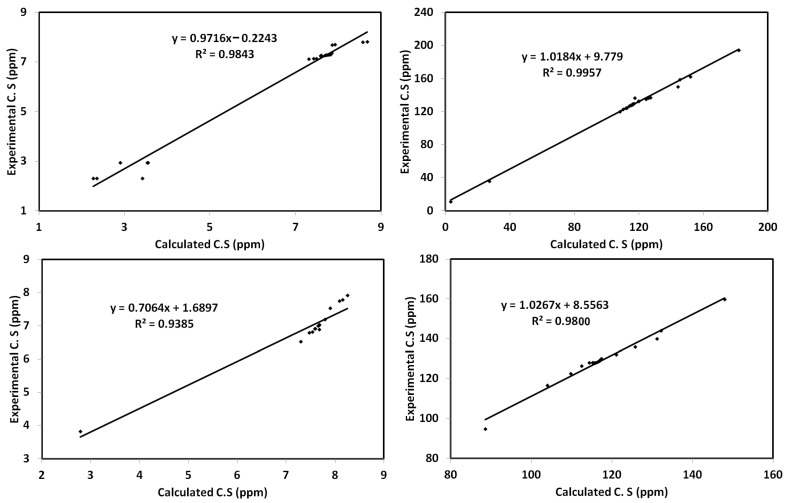
Correlations between the experimental and calculated chemical shifts for **3** (**upper**) and **4** (**lower**).

The calculated ^1^H and ^13^C{^1^H} NMR chemical shifts of **3** and **4** in chloroform as a solvent were computed using the GIAO approach. The results are depicted in Tables S3 and S4 (Supplementary Materials). Plotting the calculated ^1^H and ^13^C{^1^H} NMR chemical shifts against the experimental data gave good straight-line correlations. The correlation coefficients of these straight lines are in the range of 0.9385–0.9843 for ^1^H NMR and 0.9800–0.9957 for ^13^C{^1^H} NMR chemical shifts (Figure 10).

### 2.7. Anticancer Activity of Compounds **1**–**4**

**Figure 11:**
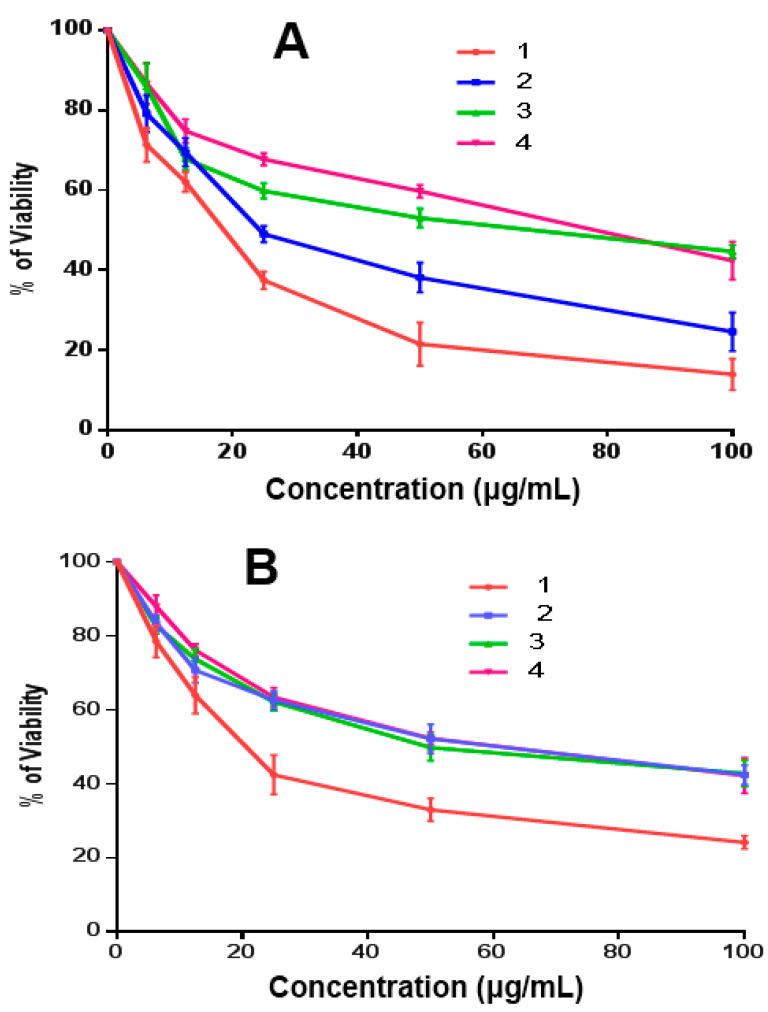
HepG2 (**A**) and MCF-7 (**B**) human cell viability assay for compounds **1**–**4** at various doses.

The obtained IC_50_ for *p*-dimethylaminobenzaldoxime **1** and *m*-nitrobenzaldoxime **2**, (4*E*)-4-(2-oxo-1,2-diphenylethylideneamino)-1,2-dihydro-1,5-dimethyl-2-phenylpyrazol-3-one **3**, and 2,3-diphenyl-2*H*-benzo[*b*][1,4]oxazin-2-ol **4** were 16.39, 27.64, 48.61, and 61.36 μg/mL against HepG2, respectively. Compound **1** had a 50% inhibitory concentration (ICs_50_) that was 1.7, 3, and 3.7 times lower than that of compounds **2**, **3**, and **4**, respectively. Compounds **1**, **2**, **3**, and **4** had ICs_50_ for MCF-7 of 22.52, 49.01, 48.46, and 53.19 μg/mL, respectively. Compound **1** IC_50_ was 2.17, 2.15, and 2.36 times lower than that of **2**, **3**, and **4**, respectively (Table 2 and Figure 11A,B). In comparison with *cisplatin* as a positive control, the IC_50_ values of the studied systems were relatively higher. Hence, the studied compounds had moderate anticancer activity, where **1** showed the most promising results against HepG2 and MCF-7 cell lines compared to the other systems.

## 3. Experimental Section

### 3.1. Chemistry

#### 3.1.1. General Methods

Reagents and solvents were obtained from commercial sources and used as received. ^1^H and ^13^C{^1^H} NMR spectra were recorded on a Bruker Avance III 400 (9.4 T, 400.13 MHz for ^1^H, 100.62 MHz for ^13^C{^1^H}) spectrometer with a 5-mm BBFO probe at 298 K. Chemical shifts (*δ* in ppm) are given relative to internal solvent CDCl_3_ 7.25 for ^1^H and 77.7 for ^13^C{^1^H}. Fourier transform infrared spectroscopy (FT-IR) spectra were recorded on an Alpha Bruker FT-IR spectrophotometer, where samples were prepared with KBr pellets, and the wavenumbers are in cm^−1^.

#### 3.1.2. Synthesis of *p*-Dimethylaminobenzaldoxime **1** and *m*-Nitrobenzaldoxime **2**

To a solution of sodium carbonate (63.38 mg, 0.598 mmol) in MeOH (10 mL), hydroxylamine hydrochloride (83.18 mg, 1.196 mmol) was added. The mixture was stirred at room temperature for 5 min. *p*-dimethylaminobenzaldehyde (162.3 mg, 1.088 mmol) or *m*-nitrobenzaldehyde (164.4 mg, 1.088 mmol) was then added, and the reaction mixture was stirred at room temperature for 12 h. The precipitate formed was then filtered off, and the filtrate was evaporated in vacuo to produce the aryl aldoximes **1** and **2**, respectively.

*p*-Dimethylaminobenzaldoxime **1**, Yield: 89%. FT-IR (cm^−1^): 3246 (OH), 1606 (C=N), 1523 (C=C). ^1^H NMR (CDCl_3_) ppm, *δ*: 2.92 (s, 6H, two C*H*_3_), 6.62 (d, *J*_HH_ 7.4 Hz, 2H, C*H*_aromatic_), 7.37 (d, *J*_HH_ 7.2 Hz, 2H, C*H*_aromatic_), 7.98 (s, 1H, C*H*=N). ^13^C{^1^H} NMR (CDCl_3_) ppm, *δ*: 39.1 (*C*H_3_), 110.7, 118.7, 127.3, 150.4 (C_aromatic_), 150.3 (*C*H=N). Anal. Calcd for C_9_H_12_N_2_O (164.2): C, 65.83; H, 7.37; N, 17.06. Found: C, 65.41; H, 7.57; N, 17.29.

*m*-Nitrobenzaldoxime **2**, Yield: 90%. FT-IR (cm^−1^): 3298 (OH), 1617 (C=N), 1535 (C=C). ^1^H NMR (CDCl_3_) ppm, *δ*: 7.51 (t, *J*_HH_ 8.0 Hz, 1H, C*H*_aromatic_), 7.83 (d, *J*_HH_ 7.7 Hz, 1H, C*H*_aromatic_), 8.14–8.17 (m, 2H, C*H*_aromatic_), 8.36 (s, 1H, C*H*=N). ^13^C{^1^H} NMR (CDCl_3_) ppm, *δ*: 120.8, 123.4, 128.8, 131.5, 132.9, 147.6 (C_aromatic_), 147.2 (*C*H=N). Anal. Calcd for C_7_H_6_N_2_O_3_ (166.13): C, 50.61; H, 3.64; N, 16.86. Found: C, 50.77; H, 3.83; N, 16.65.

#### 3.1.3. Synthesis of (4E)-4-(2-oxo-1,2-Diphenylethylideneamino)-1,2-dihydro-1,5-dimethyl-2-phenylpyrazol-3-one **3** and 2,3-diphenyl-2H-benzo[b][1,4]oxazin-2-ol **4**

To a solution of benzil (200 mg, 0.951 mmol) in EtOH (10 mL), 4-aminoantipyrine (193.3 mg, 0.951 mmol) was added, then the mixture was refluxed for 2 h. The precipitate formed was then filtered off, and the filtrate was evaporated in vacuo to produce compound **3**.

(4*E*)-4-(2-oxo-1,2-diphenylethylideneamino)-1,2-dihydro-1,5-dimethyl-2-phenylpyrazol-3-one **3**, Yield: 85%. FT-IR (cm^−1^): 1663 (C=N). ^1^H NMR (CDCl_3_), *δ*: 2.32 (s, 3H, C*H*_3_C), 2.94 (s, 3H, C*H*_3_N), 7.12–7.14 (m, 3H, C*H*_ar_), 7.25–7.31 (m, 7H, C*H*_ar_), 7.35–7.39 (m, 1H, C*H*_ar_), 7.69 (d, 2H, *J*_HH_ = 7.2 Hz, C*H*_ar_), 7.81 (d, 2H, *J*_HH_ = 7.2 Hz, C*H*_ar_). ^13^C{^1^H} NMR (CDCl_3_), *δ*: 10.99 (*C*H_3_C), 36.11 (*C*H_3_N), 120.01 (MeC=*C*), 122.83, 124.53, 126.77, 127.92, 128.35, 129.04, 129.09, 129.42, 129.92, 130.33, 132.89, 134.89 (*C*H_ar_), 136.74, 137.13 (*C*_ar_), 149.96 (Me*C*=C), 158.69 (*C*(N)=O), 162.14 (C=N), 194.46 (C=O). Anal. Calcd for C_25_H_21_N_3_O_2_ (395.45): C, 75.93; H, 5.35; N, 10.63. Found: C, 75.96; H, 5.37; N, 10.60.

To a solution of benzil (200 mg, 0.951 mmol) in EtOH (10 mL), *o*-aminophenol (103.7 mg, 0.951 mmol) was added, then the mixture was refluxed for 72 h. The precipitate formed was then filtered off, and the filtrate was evaporated in vacuo to produce compound **4**.

2,3-Diphenyl-2*H*-benzo[*b*][1,4]oxazin-2-ol **4**, Yield: 88%. FT-IR (cm^−1^): 3240 (OH), 1667 (C=N), 1589 (C=C), 1092 (C-O). ^1^H NMR (CDCl_3_), *δ*: 3.82 (bs, 1H, O*H*), 6.52 (t, 1H, *J* = 7.9 Hz, C*H*_ar_), 6.80 (t, *J* = 8.6 Hz, 2H, C*H*_ar_), 6.90 (d, 3H, *J* = 7.9 Hz, C*H*_ar_), 7.02 (t, 3H, *J* = 7.6 Hz, C*H*_ar_), 7.18–7.20 (m, 1H, C*H*_ar_), 7.52–7.54 (m, 1H, C*H*_ar_), 7.73 (d, 1H, *J* = 8.4 Hz, C*H*_ar_), 7.79 (d, 1H, *J* = 8.6 Hz, C*H*_ar_), 7.91 (d, 1H, *J* = 8.4 Hz, C*H*_ar_). ^13^C{^1^H} NMR (CDCl_3_), *δ*: 94.72 (O*C*O), 116.58, 122.60, 126.27, 127.90, 128.32, 128.89, 129.03, 129.46, 129.92, 131.95, 135.81, 139.99, 143.91 (*C*_ar_), 159.87 (C=N). Anal. Calcd for C_20_H_15_NO_2_ (301.33): C, 79.72; H, 5.02; N, 4.65. Found: C, 79.75; H, 5.04; N, 4.63.

#### 3.1.4. X-ray Structure Determinations

The X-ray diffraction data were collected on a Bruker D8 QUEST diffractometer using MoKα radiation (Table 3). The *Apex3* [46] program package was used for cell refinements and data reductions. Multi-scan absorption correction (*SADABS*) [47] was applied to the intensities before the structure solution. The structures of compounds **3** and **4** were solved by the intrinsic phasing method using the *SHELXT* [48] software. Structural refinement was carried out using *SHELXL-2017* [48].

#### 3.1.5. Methods and Calculations

##### Hirshfeld Surface Analysis

The topology analyses were performed using the Crystal Explorer 17.5 program [49].

#### 3.1.6. Computational Methods

All DFT calculations were performed using the Gaussian 09 software package [50,51] utilizing the B3LYP/6-31G(d,p) method [52,53]. Natural charges were calculated using the NBO 3.1 program as implemented in the Gaussian 09W package [54]. Structures were optimized in solutions of the compounds in CHCl_3_ using the self-consistent reaction field (SCRF) method [55,56]. Then, the NMR spectra of the studied compounds were computed using the GIAO method [57].

### 3.2. Biological Experiments

Assessment of cytotoxicity of compounds **1**–**4** against the HepG2 and MCF-7 cell lines:

The Tissue Culture Unit, Department of Biochemistry, Faculty of Science, King Abdulaziz University, provided HepG2 and MCF-7 human cell lines. In complete media, Dulbecco’s Modified Eagle’s Medium (DMEM), which contains 10% fetal bovine serum and 1% antibiotic, was used to cultivate the human cell lines at 37 °C and 95% humidity in a 5% CO_2_ incubator for 24–48 h. After 70–90% of the confluent cells were completed, the cells were gathered, then 4 mL of 0.25% trypsin with EDTA were incubated in a CO_2_ incubator for 5 min. The addition of 5 mL of the complete medium was made to stop the activity of the trypsin process. Unattached cell-containing medium was centrifuged; the pellets were then twice washed in sterile phosphate buffer saline (PBS). After 20 μL of this cell-containing media were stained with 20 μL of 0.4% trypan blue, the numbers of cells were counted using a hemocytometer in the four major squares. The following equation was used to compute the number of cells per milliliter:

1/4 × 10^4^ × 2

A 96-well microplate was filled with 0.1 mL of 5000 cells suspended in complete media, and the plate was then cultured in the incubator for 24 h. Anticancer effects of compounds **1**–**4** were assessed. The *cisplatin* was used as a positive control against the two human cell lines. Each compound was added to the media at different concentrations, ranging from 6.25 to 100 μg/mL and 3.125 to 50 μg/mL for *cisplatin*, once 70% of the cells in each well had reached confluence. The 96-well plates were placed in the incubator for 48 h before the media in each well was changed to 100 μL of free media with 0.5 mg of MTT/mL for 4 h. Each well received 100 μL of dimethylsulfoxide (DMSO), which was added and kept at room temperature for 15 min before being detected by a microplate reader at 595 nm (Bio-RAD microplate reader, Japan). The 50% inhibitory concentrations (ICs_50_) of **1**, **2**, **3**, and **4** treated with two attached cell lines were computed using the GraphPad Prism program version 9, utilizing the curve of cell viability vs. various concentrations [58,59].

## 4. Conclusions

In conclusion, we succeeded in synthesizing *p*-dimethylaminobenzaldoxime **1** and *m*-nitrobenzaldoxime **2**, in excellent yields, by treatment of *p*-dimethylaminobenzaldehyde or *m*-nitrobenzaldehyde, respectively, with sodium carbonate and hydroxylamine hydrochloride in MeOH at room temperature. Moreover, (4*E*)-4-(2-oxo-1,2-diphenylethylideneamino)-1,2-dihydro-1,5-dimethyl-2-phenylpyrazol-3-one **3** and the unexpected 2,3-diphenyl-2*H*-benzo[*b*][1,4]oxazin-2-ol **4** were synthesized, in very good yields, by treatment of benzil with 4-aminoantipyrine or *o*-aminophenol, respectively, in refluxing EtOH. Compounds **1**–**4** were characterized by FT-IR, ^1^H, and ^13^C{^1^H} NMR spectroscopy elemental analyses. In addition, the structures of **3** and **4** were confirmed using single crystal X-ray diffraction. Analysis of molecular packing with the aid of Hirshfeld calculations is presented. The different intermolecular interactions that control the crystal stability of the studied systems were analyzed based on qualitative and quantitative levels. In **3**, the O…H (11.1%), N…H (3.4%), C…H (29.4%), and C…C (1.6%) contacts are the most important, while in **4**, the O…H (8.8%), N…H (5.7%), and C…H (30.3%) contacts have the upper hand in controlling the crystal stability. Different electronic and geometric parameters of **3** and **4** were calculated at their optimized structures using the DFT/B3LYP/6-31G(d,p) method. In addition, the calculated NMR spectra were in good agreement with the experimental data. The ICs_50_ of compounds **1**, **2**, **3**, and **4** for HepG2 were 16.39, 27.64, 48.61, and 61.36 μg/mL, respectively. The concentrations of **1** that suppressed HepG2 growth by 50% (IC_50_) were 1.7, 3, and 3.7 times lower than those of **2**, **3**, and **4**, respectively. The ICs_50_ for MCF-7 of **1**, **2**, **3**, and **4** were 22.52, 49.01, 48.46, and 53.19 μg/mL, respectively. The IC_50_ of **1** was 2.17, 2.15, and 2.36 times lower than those of **2**, **3**, and **4**, respectively. Hence, compound **1** exhibits the most promising anticancer activity with a modest anticancer potential when compared to *cisplatin* as positive control.

## Figures and Tables

**Table 1:** The short intermolecular interactions in **3** and **4**.

Contact	Distance	Contact	Distance	Contact	Distance
3				4	
O1…H2	2.420	C15…H19	2.748	O2…H20	2.284
O1…H25B	2.557	C6…H23	2.741	N1…H1	2.028
O2…H21	2.489	C7…H23	2.775	C1…H3	2.759
N1…H23	2.482	C16…C19	3.350	C6…H3	2.718
C11…H24B	2.608				

**Table 2:** IC_50_ values of compounds **1**–**4** in HepG2 and MCF-7.

Compound					
	1	2	3	4	*Cisplatin*
HepG2	14.51–18.51 *	24.67–30.96 *	37.85–62.42 *	51.39–73.26 *	1.767–2.988 *
	16.39 **	27.64 **	48.61 **	61.36 **	2.297 **
MCF-7	19.67–25.79 *	40.00–60.03 *	40.15–58.49 *	45.77–61.82 *	3.781–6.837 *
	22.52 **	49.01 **	48.46 **	53.19 **	5.085 **

* Range ** Value.

**Table 3:** Crystal data and details of the structure determination of **3** and **4**.

Compound	3	4
CCDC	2245159	2245160
Formula	C_25_H_21_N_3_O_2_	C_20_H_15_NO_2_
Formula Weight	395.45	301.33
Crystal System	Monoclinic	Orthorhombic
Space Group	P21/c	Pbca
a, b, c [Å]	8.253 (3)	8.202 (4)
	28.476 (12)	12.001 (5)
	8.776 (4)	31.351 (13)
alpha, beta, gamma [°]	90	
	96.841 (13)	
	90	
V [Å^3^]	2047.8 (15)	3086 (2)
Z	4	8
D(calc) [g/cm^3^]	1.283	1.297
Mu(MoKa) [/mm ]	0.083	0.084
F(000)	832	1264
Crystal Size [mm]	0.07 × 0.15 × 0.22	0.15 × 0.19 × 0.22
Data Collection		
Temperature (K)	293	289
Radiation [Å]	MoKa	MoKa
	0.71073	0.71073
Theta Min-Max [°]	2.4, 25.4	2.6, 25.4
Dataset	−9: 9; −34: 34; −10: 10	−9: 9; −13: 14; −37: 37
Tot., Uniq. Data, R(int)	18671, 3750, 0.069	30981, 2825, 0.138
Observed Data [I > 2.0 sigma(I)]	2758	1795
Refinement		
Nref, Npar	3750, 274	2825, 211
R, wR2, S	0.0849, 0.2321, 1.08	0.0510, 0.1118, 1.03
	w = ^2^(FO^2^) + (0.0699P)^2^ + 4.3819P] WHERE P = (FO^2^+2FC^2^)/3′	w = ^2^(FO^2^) + (0.0537P)^2^ + 0.1289P] WHERE P = (FO^2^+2FC^2^)/3′
Min. and Max. Resd. Dens. [e/Å^3^]	−0.33, 0.32	−0.21, 0.16

## Data Availability

Not available.

## Supplementary Materials

The following supporting information can be downloaded at https://www.mdpi.com/article/10.3390/molecules28124766/s1: Table S1. Cartesian coordinates for the calculated structure of **3**; Table S2. Cartesian coordinates for the calculated structure of **4**; Table S3. NMR chemical shifts for **3**; Table S4. NMR chemical shifts for **4**; Figure S1. ^1^H NMR spectrum of **1** in CDCl_3_; Figure S2. ^13^C{^1^H} NMR spectrum of **1** in CDCl_3_; Figure S3. ^1^H NMR spectrum of **2** in CDCl_3_; Figure S4. ^13^C{^1^H} NMR spectrum of **2** in CDCl_3_; Figure S5. FT-IR spectrum of **1**; Figure S6. FT-IR spectrum of **2**; Figure S7. ^1^H NMR spectrum of **3** in CDCl_3_; Figure S8. ^13^C{^1^H} NMR spectrum of **3** in CDCl_3_; Figure S9. ^1^H NMR spectrum of **4** in CDCl_3_; Figure S10. ^13^C{^1^H} NMR spectrum of **4** in CDCl_3_; Figure S11. FT-IR spectrum of **3**; Figure S12. FT-IR spectrum of **4**.
