# Supplementary material for: Synthesis, Crystal Structure, DFT, and Anticancer Activity of Some Imine-Type Compounds via Routine Schiff Base Reaction: An Example of Unexpected Cyclization to Oxazine Derivative
Source: Molecules. 2023 Jun 14;28(12):4766. doi: 10.3390/molecules28124766 (PMC10301602; doi:10.3390/molecules28124766)

Supplementary Data

Synthesis, crystal structure, DFT and anticancer activity of some imine-type compounds *via* routine Schiff base reaction: An example of unexpected cyclization to oxazine derivative

Jamal Lasri [1,*], Naser E. Eltayeb [1,*], Saied M. Soliman [2,*], Ehab M. M. Ali [3], Sultan Alhayyani [1], Abdullah Akhdhar [4]

[1] *Department of Chemistry, Rabigh College of Science and Arts, P.O. Box 344, King Abdulaziz University, Jeddah 21589, Saudi Arabia*

[2] *Department of Chemistry, Faculty of Science, Alexandria University, Ibrahimia, Alexandria 21321, Egypt*

[3] *Department of Biochemistry, Faculty of Science, King Abdulaziz University, Jeddah 21589, Saudi Arabia*

[4] *Department of Chemistry, College of Science, University of Jeddah, Jeddah, Saudi Arabia*

* Corresponding authors.

E-mail addresses: jlasri@kau.edu.sa, netaha@kau.edu.sa, saeed.soliman@alexu.edu.eg

Table S1. Cartesian coordinates for the calculated structure of **3**.

Table S2. Cartesian coordinates for the calculated structure of **4**.

Table S3. NMR chemical shifts for **3**.

Table S4. NMR chemical shifts for **4**.

Figure S1. ^{1}H NMR spectrum of **1** in $CDCl_3$.

Figure S2. $^{13}C\{^{1}H\}$ NMR spectrum of **1** in $CDCl_3$.

Figure S3. ^{1}H NMR spectrum of **2** in $CDCl_3$.

Figure S4. $^{13}C\{^{1}H\}$ NMR spectrum of **2** in $CDCl_3$.

Figure S5. FT-IR spectrum of **1**.

Figure S6. FT-IR spectrum of **2**.

Figure S7. ^{1}H NMR spectrum of **3** in $CDCl_3$.

Figure S8. $^{13}C\{^{1}H\}$ NMR spectrum of **3** in $CDCl_3$.

Figure S9. ^{1}H NMR spectrum of **4** in $CDCl_3$.

Figure S10. $^{13}C\{^{1}H\}$ NMR spectrum of **4** in $CDCl_3$.

Figure S11. FT-IR spectrum of **3**.

Figure S12. FT-IR spectrum of **4**.

Table S1. Cartesian coordinates for the calculated structure of **3**.

O	-0.771478000	0.553378000	1.956593000
O	0.928649000	0.863779000	-0.629474000
N	-0.871486000	-1.655184000	-0.120258000
N	2.630452000	-0.730312000	-0.442532000
N	2.687135000	-2.103754000	-0.106065000
C	-2.490685000	4.337235000	-0.874233000
H	-2.784005000	5.290668000	-1.304519000
C	-1.948711000	4.288560000	0.413357000
H	-1.818397000	5.203777000	0.983516000
C	-1.572681000	3.067103000	0.963416000
H	-1.141645000	3.002822000	1.956865000
C	-1.740632000	1.881918000	0.233403000
C	-1.329406000	0.597594000	0.870030000
C	-1.713694000	-0.743473000	0.228654000
C	0.500473000	-1.498780000	-0.169895000
C	1.286865000	-0.291610000	-0.434459000
C	3.735694000	0.105981000	-0.129299000
C	4.663516000	-0.261864000	0.853807000
H	4.518704000	-1.181384000	1.409958000
C	5.747106000	0.572220000	1.126858000
H	6.464126000	0.280413000	1.888678000
C	5.900133000	1.779737000	0.445050000
H	6.741483000	2.429485000	0.665447000
C	-2.653227000	3.161099000	-1.609542000
H	-3.068093000	3.198815000	-2.612405000
C	-2.282016000	1.936821000	-1.057849000
H	-2.404566000	1.025289000	-1.633738000
C	-3.159779000	-1.083427000	0.181221000
C	-3.602557000	-2.157655000	-0.611374000
H	-2.869136000	-2.708524000	-1.189474000
C	-4.949293000	-2.502381000	-0.649691000
H	-5.276043000	-3.330935000	-1.271680000
C	-5.881875000	-1.784299000	0.104997000
H	-6.933623000	-2.053557000	0.073028000
C	-5.454780000	-0.718818000	0.897562000
H	-6.171192000	-0.159066000	1.491453000
C	-4.106271000	-0.366506000	0.932932000
H	-3.790358000	0.460598000	1.559850000
C	1.374015000	-2.549863000	-0.058725000
C	1.040317000	-3.992710000	0.113298000
H	1.405998000	-4.600160000	-0.722581000
H	-0.044932000	-4.088833000	0.163873000
H	1.473239000	-4.397601000	1.034622000
C	3.677693000	-2.869744000	-0.873139000
H	3.710785000	-3.884552000	-0.474860000
H	4.659145000	-2.414558000	-0.740425000
H	3.433151000	-2.898279000	-1.942658000
C	4.959511000	2.148324000	-0.518800000
H	5.065819000	3.089061000	-1.050807000
C	3.883701000	1.315746000	-0.820221000
H	3.148970000	1.596769000	-1.563250000

Table S2. Cartesian coordinates for the calculated structure of **4**.

O	-1.406610000	1.299074000	0.132544000
O	-0.271185000	0.577804000	2.029147000
H	-0.414697000	1.481814000	2.351035000
N	-1.239567000	-1.467659000	-0.187894000
C	-2.475979000	-0.824300000	-0.133833000
C	-3.658770000	-1.559670000	-0.289583000
H	-3.576382000	-2.633956000	-0.420017000
C	-4.892616000	-0.915858000	-0.276367000
H	-5.806430000	-1.489756000	-0.392543000
C	-4.951911000	0.474406000	-0.123230000
H	-5.913019000	0.979851000	-0.120680000
C	-3.784591000	1.223047000	0.030168000
H	-3.808831000	2.301629000	0.146345000
C	-2.557838000	0.564730000	0.036036000
C	-0.222665000	0.643620000	0.623722000
C	0.913116000	1.520619000	0.094089000
C	0.998850000	1.774021000	-1.280811000
H	0.264940000	1.339207000	-1.951817000
C	2.010322000	2.586646000	-1.786797000
H	2.067192000	2.773375000	-2.855098000
C	2.944801000	3.164115000	-0.923609000
H	3.732289000	3.799457000	-1.318032000
C	2.861306000	2.919802000	0.445984000
H	3.583009000	3.364518000	1.124903000
C	1.851478000	2.098439000	0.952112000
H	1.802284000	1.900042000	2.017658000
C	-0.171709000	-0.827969000	0.143519000
C	1.120295000	-1.554345000	0.076839000
C	2.154687000	-1.326490000	0.998238000
H	2.018899000	-0.596479000	1.786684000
C	3.335202000	-2.064648000	0.931043000
H	4.121984000	-1.885164000	1.657944000
C	3.505018000	-3.033117000	-0.059083000
H	4.429120000	-3.601299000	-0.113923000
C	2.478528000	-3.271716000	-0.976319000
H	2.602167000	-4.025953000	-1.748120000
C	1.294235000	-2.544474000	-0.905161000
H	0.486248000	-2.729837000	-1.604347000

Table S3. NMR chemical shifts (C.S) for **3**.

Atom	C.S(calc)	C.S(exp)	Atom	C.S(calc)	C.S(exp)
C6	119.839	132.887	H7	7.823	7.308
C8	115.620	128.356	H9	7.850	7.365
C10	116.316	129.042	H11	8.575	7.803
C12	126.056	136.738	H19	7.420	7.139
C13	182.059	194.462	H21	7.779	7.295
C14	152.046	162.143	H23	7.583	7.251
C15	110.180	122.833	H25	7.605	7.269
C16	145.596	158.694	H27	7.919	7.702
C17	124.556	134.891	H30	8.679	7.821
C18	108.309	120.01	H32	7.853	7.685
C20	116.388	129.418	H34	7.711	7.281
C22	112.589	124.536	H36	7.493	7.142
C24	115.028	127.924	H38	7.313	7.119
C26	119.974	132.887	H41	2.353	2.319
C28	127.335	137.129	H42	3.416	2.319
C29	113.943	126.769	H43	2.269	2.319
C31	115.978	128.356	H45	3.530	2.939
C33	116.980	129.918	H46	3.546	2.939
C35	115.119	127.924	H47	2.902	2.939
C37	117.496	136.326	H49	7.687	7.275
C39	144.263	149.959	H51	7.758	7.291
C40	3.180	10.989			
C44	27.255	36.107			
C48	116.361	129.087			
C50	112.070	124.536			

Table S4. NMR chemical shifts (C.S) for **4**.

Atom	C.S(calc)	C.S(exp)	Atom	C.S(calc)	C.S(exp)
C5	121.105	131.948	H3	2.787	3.823
C6	116.967	129.027	H7	7.905	7.538
C8	109.802	122.601	H9	7.541	6.811
C10	117.107	129.459	H11	7.676	6.887
C12	103.992	116.58	H13	7.302	6.52
C14	132.208	143.914	H18	7.681	7.039
C15	88.609	94.723	H20	7.599	6.91
C16	131.212	139.995	H22	7.657	7
C17	116.516	128.322	H24	7.794	7.197
C19	115.955	127.899	H26	8.158	7.79
C21	116.007	127.899	H30	8.091	7.747
C23	115.206	127.899	H32	7.472	6.793
C25	112.449	126.272	H34	7.591	6.907
C27	147.966	159.869	H36	7.678	7.016
C28	125.823	135.809	H38	8.258	7.916
C29	117.377	129.922			
C31	114.360	127.899			
C33	116.829	128.89			
C35	115.466	127.899			
C37	116.979	129.027			

Figure S1. ^{1}H NMR spectrum of **1** in $CDCl_3$.

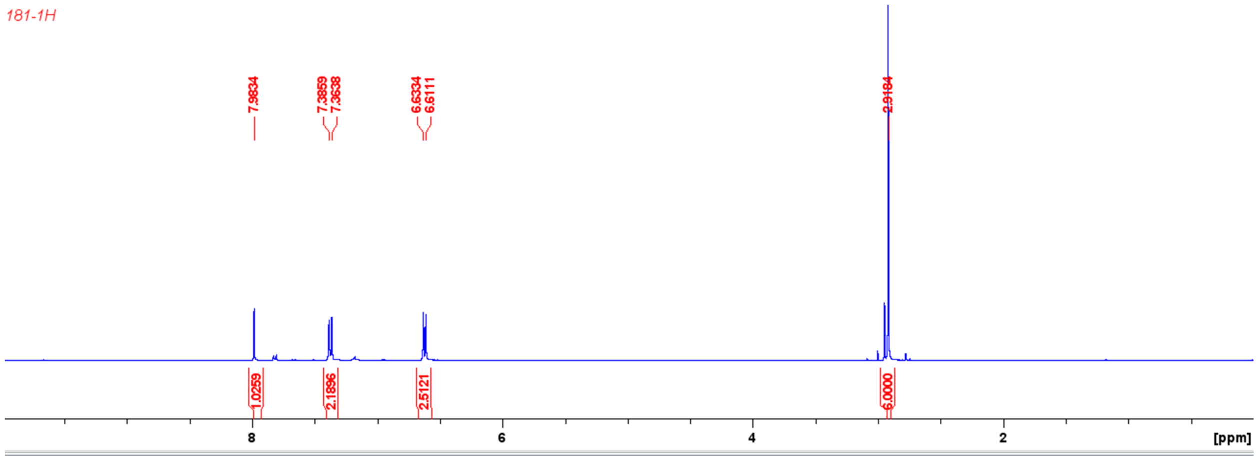

Figure S2. $^{13}C\{^{1}H\}$ NMR spectrum of **1** in $CDCl_3$.

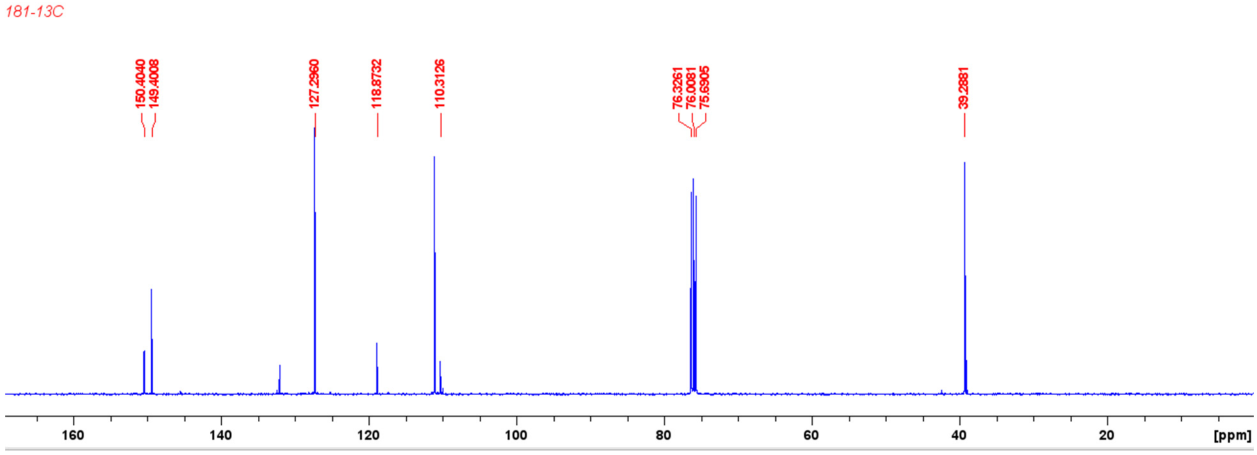

Figure S3. ^{1}H NMR spectrum of **2** in $CDCl_3$.

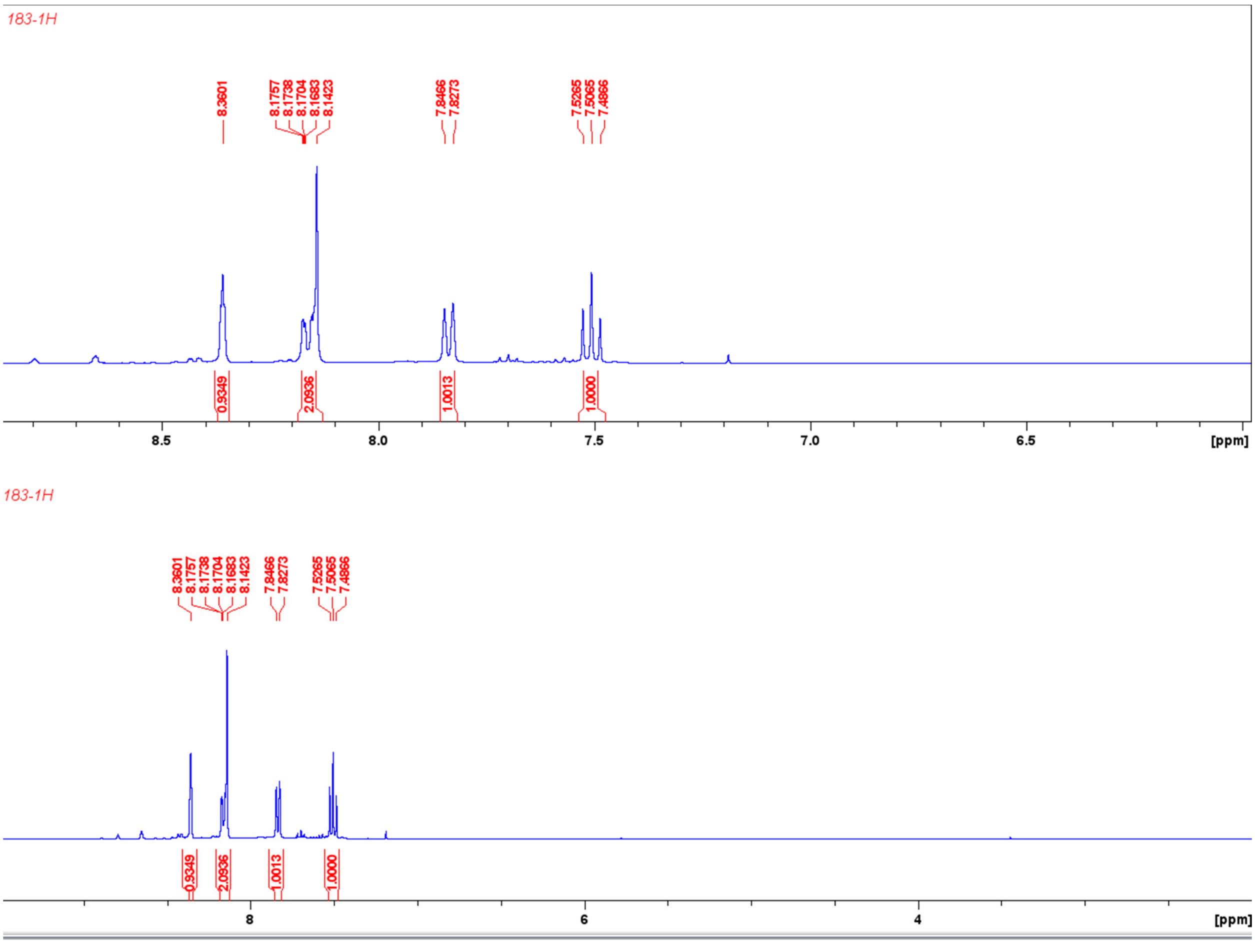

Figure S4. $^{13}C\{^{1}H\}$ NMR spectrum of **2** in $CDCl_3$.

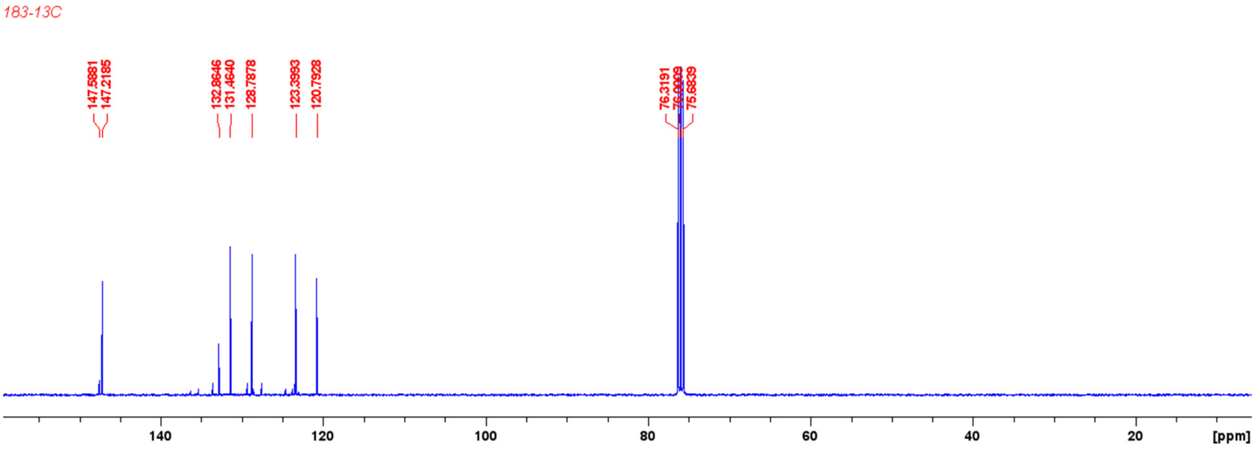

Figure S5. FT-IR spectrum of **1**.

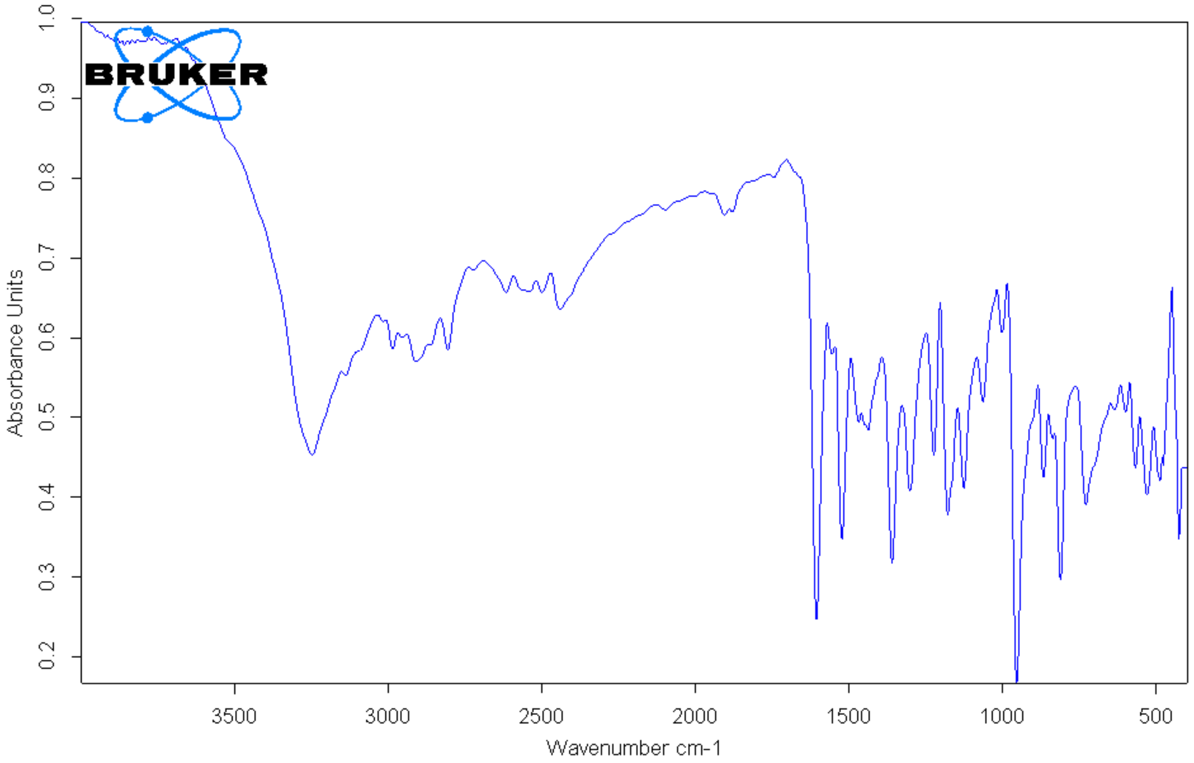

Figure S6. FT-IR spectrum of **2**.

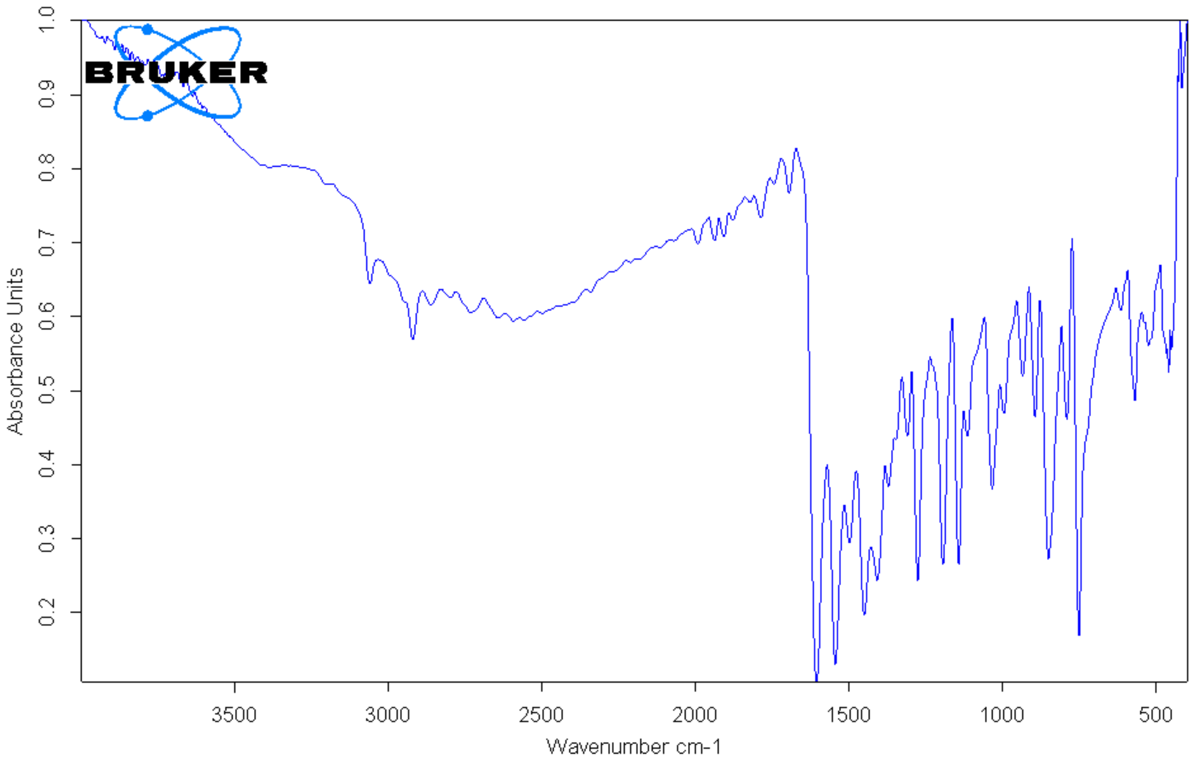

Figure S7. ^{1}H NMR spectrum of **3** in $CDCl_3$.

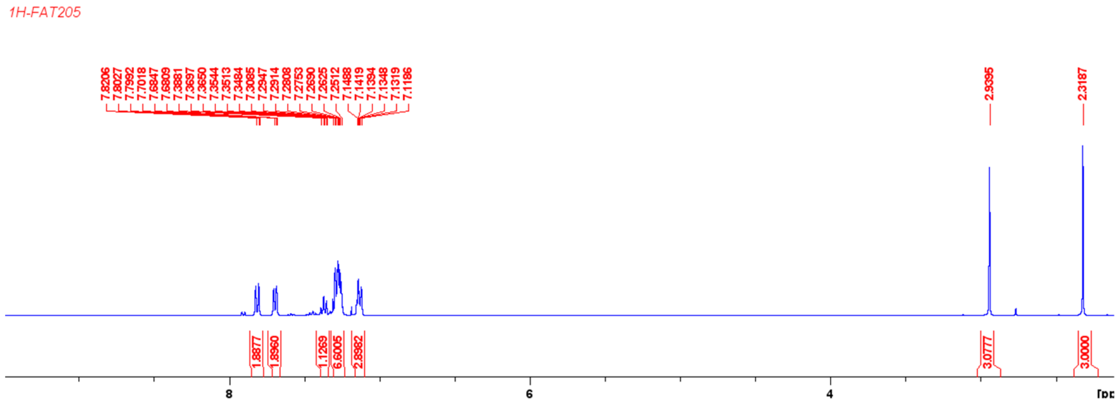

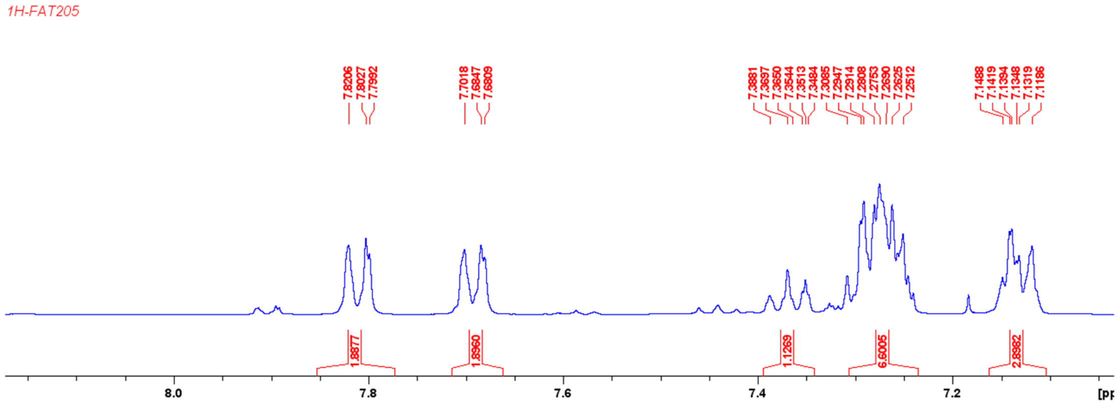

Figure S8. $^{13}C\{^{1}H\}$ NMR spectrum of **3** in $CDCl_3$.

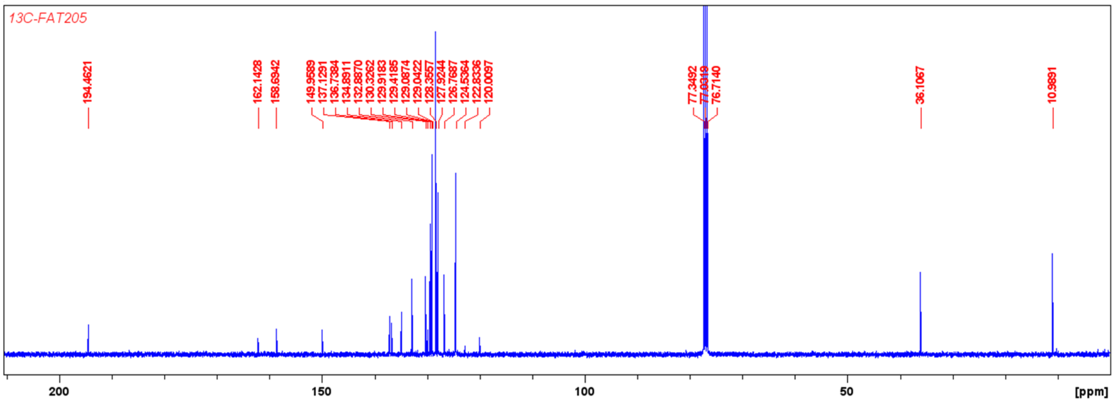

Figure S9. ^{1}H NMR spectrum of **4** in $CDCl_3$.

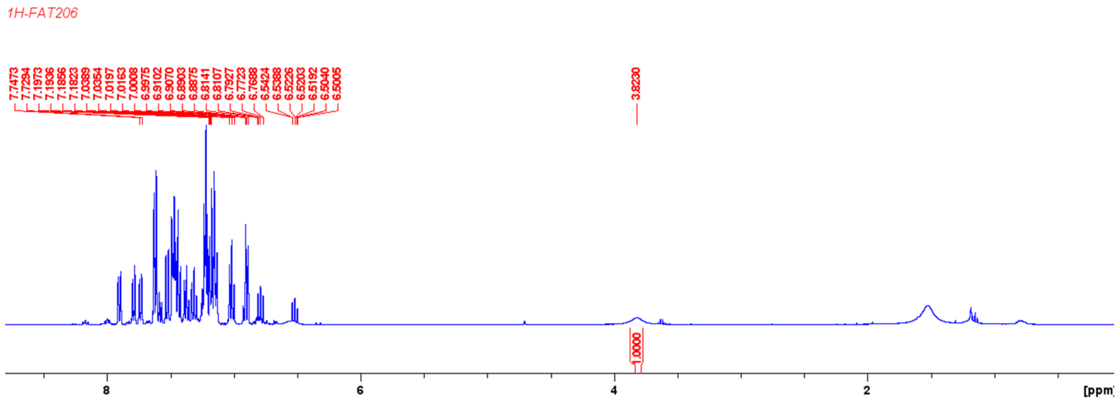

Figure S10. $^{13}C\{^{1}H\}$ NMR spectrum of **4** in $CDCl_3$.

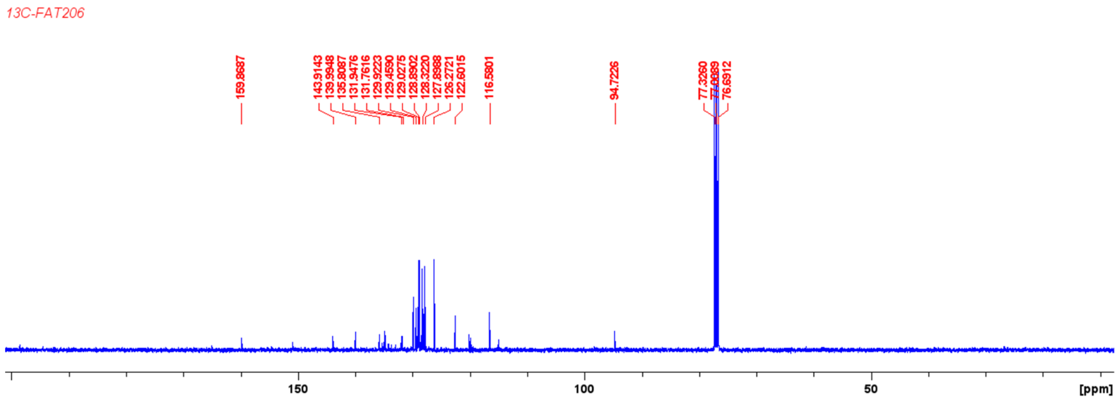

Figure S11. FT-IR spectrum of **3**.

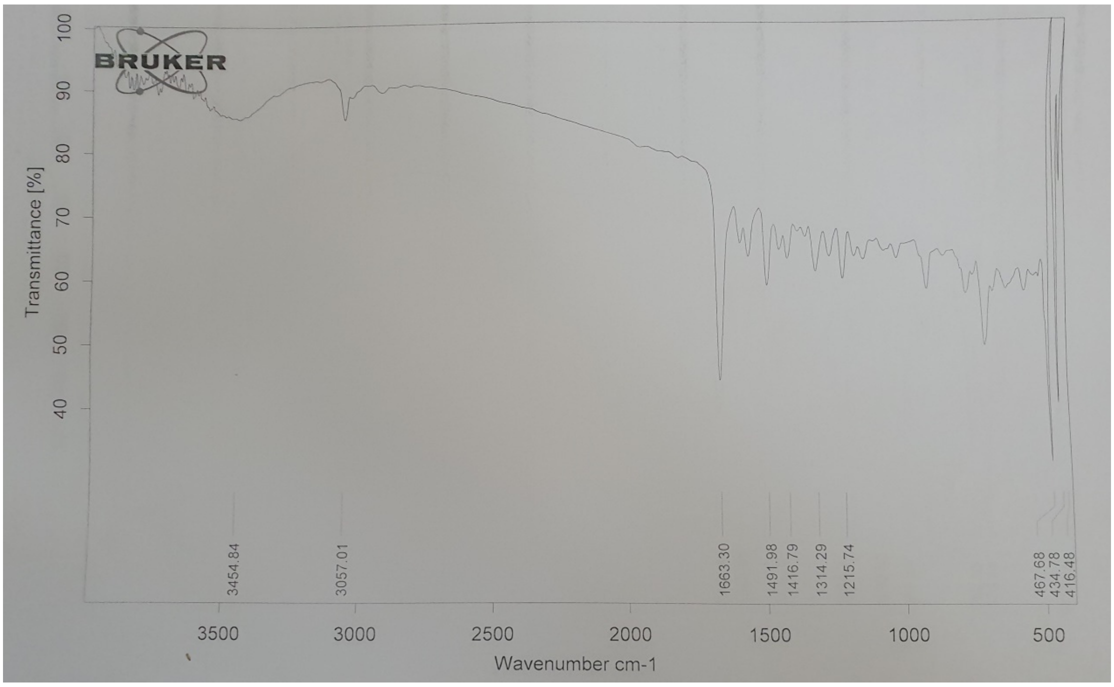

Figure S12. FT-IR spectrum of **4**.

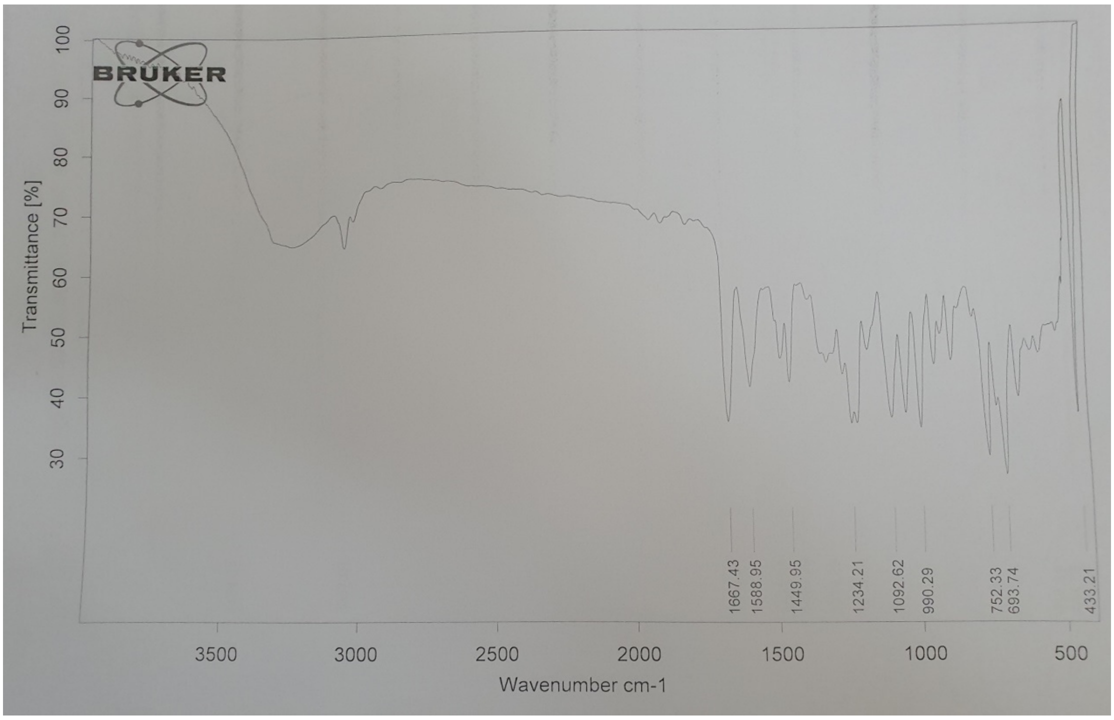

Supplement: Supplementary file 1 [file molecules-28-04766-s001.zip › molecules-2335712-supplementary.pdf]
